# The diffuse involvement of anti-N-methyl-D-aspartate receptor encephalitis in brain: a case report

**DOI:** 10.1186/s12883-019-1456-6

**Published:** 2019-09-26

**Authors:** Yun Jiang, Jianpeng Ma, Tao Gong, Hongjun Hao, Haibo Chen

**Affiliations:** 1 Department of Neurology, Beijing Hospital, National Center of Gerontology, Beijing, China; 2 Department of Neurology, Beijing Friendship Hospital, Beijing, China; 3 Department of Neurology, Peking University First Hospital, Beijing, China

**Keywords:** Anti-N-methyl-D-aspartate receptor (NMDAR) encephalitis, Clinic, Brain, Magnetic resonance image

## Abstract

**Background:**

Anti-N-methyl-D-aspartate receptor (NMDAR) encephalitis is a severe and most common autoimmune encephalitis in patients under 40 years old. Anti-NMDAR encephalitis has various clinical and neuroimaging findings. Here we report a special case of an anti-NMDAR encephalitis who had diffuse lesions in bilateral hemispheres with mild mass effects in left basal ganglia area.

**Case presentations:**

A 28-year-old female anti-NMDAR encephalitis patient mainly presented with headache and fever. Brain magnetic resonance image (MRI) showed slightly contrasted diffuse lesions, involving the left temporal and frontal lobes, left basal ganglia area and splenium of corpus callosum, as well as the right frontal lobe, with mild edema surrounded in the left basal ganglia area. Cerebrospinal fluid (CSF) revealed a moderate pleocytosis with normal protein and glucose levels. Anti-NMDAR antibodies were identified in CSF. Transvaginal ovarian ultrasound did not reveal an ovarian teratoma. The patient was treated with immunoglobulin and steroid, and had a good recovery.

**Conclusions:**

Anti-NMDAR encephalitis has no special clinical manifestations and brain MRI is highly variable, which could be unremarkable or abnormal involving white and grey matters. The extensive lesions in frontal and temporal lobes, and basal ganglia area, with mild mass effects, have not been described previously. Recognition of various changes in brain MRI will enable the early detection of anti-NMDAR antibody and then effective treatments.

**Electronic supplementary material:**

Supplementary information accompanies this papaer at 10.1186/s12883-019-1456-6.

## Background

Anti-N-methyl-D-aspartate receptor (NMDAR) encephalitis is a severe autoimmune disorder first described in 2007 [1]. It is associated with antibodies in the serum and cerebrospinal fluid against the GluN1 subunit of the NMDAR [2, 3]. This disease has a female predominance and up to 50% of cases have ovarian teratoma [4]. It may also be associated with other tumors, such as lung or breast carcinoma [5]. In addition, some studies demonstrated that virus [6] may induce anti-NMDAR encephalitis. Clinically, anti-NMDAR encephalitis is characterized by severe psychiatric symptoms, memory loss, seizure, dyskinesia, and autonomic instability [4, 7].

According to previous reports, brain magnetic resonance image (MRI) is unremarkable in about 50% anti-NMDAR encephalitis patients, while brain MRI in the other half is highly variable in white and grey matter [3, 4, 8–10]. Here we present a special case of an anti-NMDAR encephalitis with diffuse lesions in the left frontal and temporal lobes, the left basal ganglia area as well as right frontal lobe on brain MRI. Mild edema was present around the lesions in the left basal ganglia area. Diffuse cerebral glioma was highly suspected on admission and anti-NMDAR encephalitis was diagnosed once anti-NMDAR antibodies in CSF were identified. The patient accepted immunoglobulin and steroid treatments and had a good outcome.

## Case presentations

A 28-year-old previously healthy female presented with headache and fever. At onset, she had intermittent distended headaches predominantly on the left side and she occasionally took oral ibuprofen. One week later, the headaches became consistent and intolerable, accompanied by severe nausea and vomiting. She developed a fever around 38 °C. Blood routine test showed WBC 18.52*10^9^/L, N 80.6%. She did not present influenza or diarrhea. She was treated with antibiotics for a week, but her symptoms exacerbated. She was transferred to our hospital nineteen days after the symptom onset. On admission, she was apathic and physical examinations did not reveal focal neurological deficits. Routine blood test indicated WBC 10.83*10^9^/L, N 78.1%. The erythrocyte sedimentation rate was normal. C-reactive protein and procalcitonin levels were normal. Blood chemistry analysis including liver and renal functions as well as creatine kinase level, were normal. Coagulation and D-Dimer were normal. Autoimmune markers including antinuclear antibodies (ANA), anti-double-stranded DNA antibodies (dsDNA), SSA and SSB antibodies, and anti-neutrophil cytoplasmic antibodies (ANCA) were all negative. Blood thyroid function tests presented normal thyroid function but increased levels of anti-thyroid peroxidase antibodies (233 IU/ml, normal range 0–70) and anti-thyroglobulin antibodies (147.1 IU/ml, normal range 0–70). Serum tumor markers were all within normal range.

**Fig. 1:**
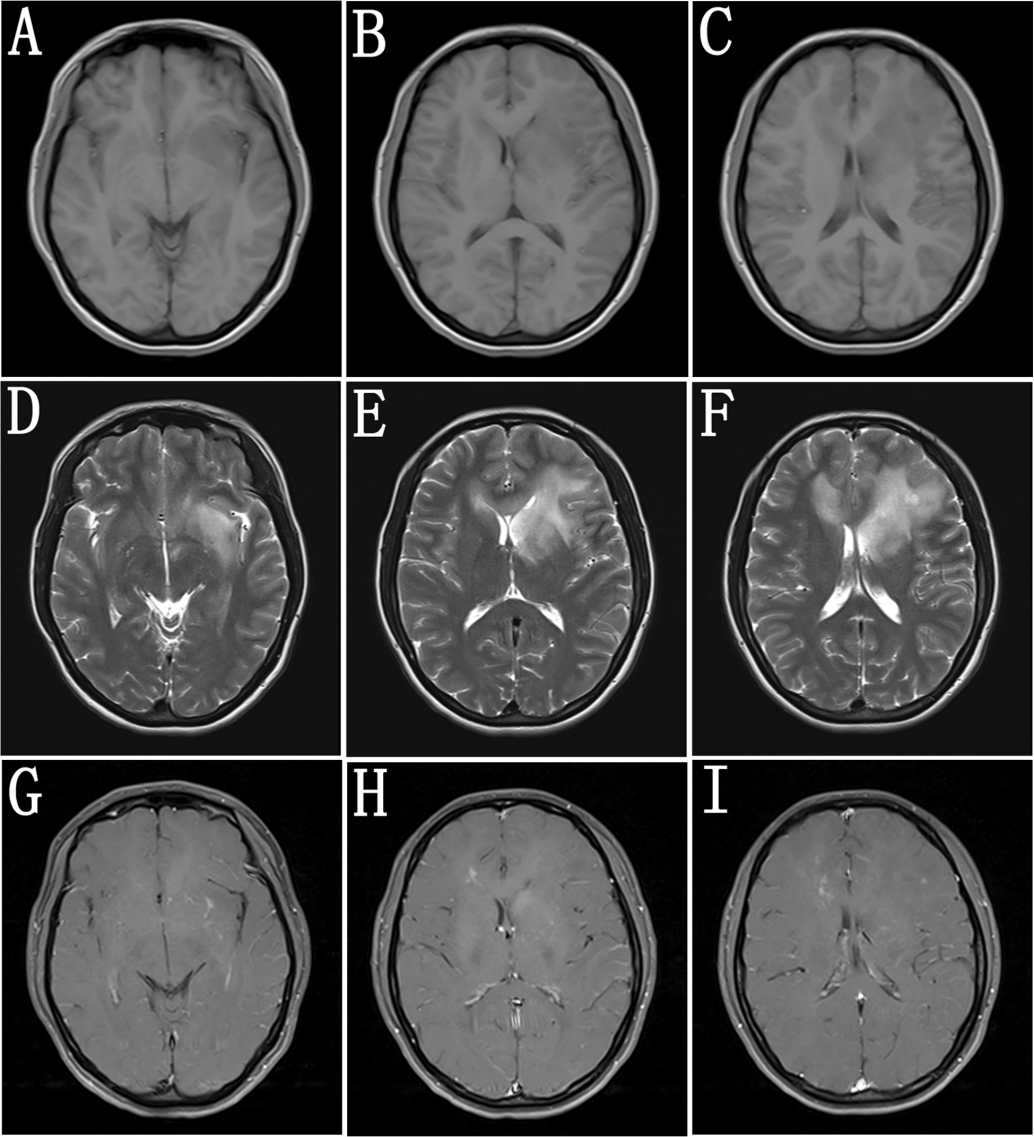
Brain MRI before treatments. Ten days after symptom onset, brain MRI revealed diffuse lesions in the left temporal and frontal lobes, the left basal ganglia area, the left splenium of corpus callosum, and the right frontal lobe, which were hypointensity on T1WI (**a**-**c**), and hyperintensity on T2WI (**d**-**f**). The lesion in the left basal ganglia area had mild edema and the anterior of left lateral ventricule was compressed (**b**, **e**, **h**). Barely contrasted lesions were present in the above lesions (**g**-**i**)

Brain MRI presented T1WI hypointense (Fig. 1 a-c), T2WI (Fig. 1 d-f) and FLAIR hyperintense lesions in the left frontal and temporal lobes, the left basal ganglia area, the left splenium of corpus callosum, and the right frontal lobe with mild edema surrounding in the left basal ganglia area. No apparent enhancement was revealed in the lesions (Fig. 1 g-i). Electroencephalography (EEG) showed widespread slow waves with higher amplitude.

**Fig. 2:**
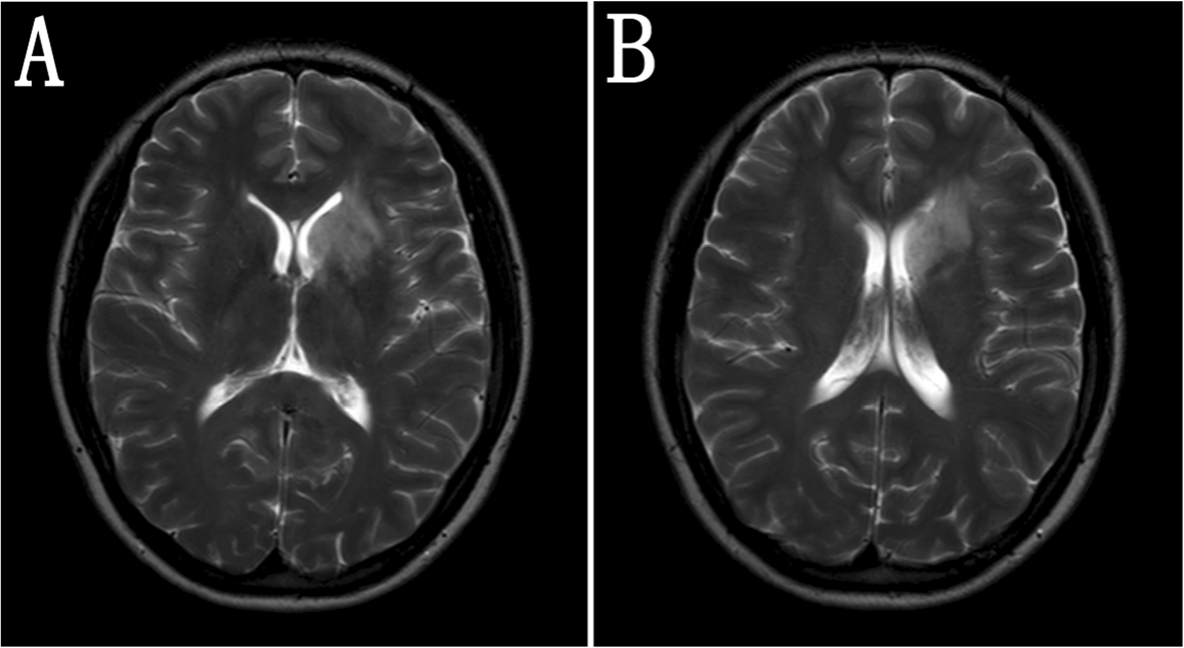
Brain MRI after treatments. Forty days after the initiation of treatments, brain MRI showed that the lesion in right frontal lobe nearly disappeared, and the lesions in left frontal lobe and left basal ganglia and callosum were reduced (**a**, **b**)

Cerebrospinal fluid (CSF) analysis showed a moderate pleocytosis (116*10^6^/L) with mononuclear cell predominance (98.2%), and normal protein and glucose levels. CSF cytology did not reveal tumor cells. CSF oligoclonal IgG bands were positive and the IgG index was elevated (14.12 mg/24 h, normal range < 7.0 mg/24 h). The CSF antibody against myelin oligodendrocyte glycoprotein (MOG) was negative. Serum antibody against aquaporin-4 (AQP-4) was negative. The serum and CSF were negative for paraneoplastic autoantibodies, including Hu, Ri, Yo, amphiphysin, CV2 and Ma2, as well as for autoimmune encephalitis-related antibodies such as alpha-amino-3-hydroxy-5-methyl-4-isoxazolepropionic acid receptor (AMPAR), gamma-aminobutyric acid beta receptor (GABA_B_R), contactin-associated protein-like 2 (CASPR2), and leucine-rich glioma inactivated protein 1 (LGI1). Anti-NMDAR antibodies however, were identified in CSF (1:32), using a cell based assay via indirect immunofluorescence on HEK293 cells transfected with homodimers of the NR1a subunits of the NMDAR (Euroimmun Medizinische Labordiagnostika AG, Lübeck, Germany) (Neuroimmunal lab, Peking University First Hospital, Beijing, China) (Additional files 1 and 2). CSF extensive studies for viral, bacterium and fungi were negative. Diagnosis of anti-NMDAR encephalitis was confirmed. Transvaginal ovarian ultrasound did not reveal an ovarian teratoma. Furthermore, tumor screening by whole body imaging, including computed tomography of the chest, abdomen, and pelvis did not indicate abnormalities. ^18^F-fluorodeoxyglucose (FDG) positron emission tomography (PET) showed hyperactivity in cortex of the left frontal, temporal, parietal lobes and right cerebellum with the most predominance exhibited in the left frontal lobe cortex, hypoactivity in the frontal of left basal ganglia, and no potential neoplasia alterations outside the brain. Cognitive function tests of Montreal cognitive assessment disclosed immediate memory impairment with a memory quotient value of 89. She received 2 rounds of immunoglobulin and high dose of steroid treatments. Immunoglobulin, 0.4 g/kg/day, was given intravenously for 5 days, followed by steroid pulse therapy with methylprednisolone, 1 g/day, for 3 days. The dosage of methylprednisolone was continuously cut half every 3 days until 120 mg, and then replaced by oral prednisone 60 mg/day, which was subsequently tapered off. Ten days after the treatments, the patient’s headache disappeared and she recovered to normal appetite. Another 2 weeks later, her body temperature was completely contained within normal range. Six weeks after admission, brain MRI showed that the lesions in bilateral hemispheres were markedly reduced (Fig. 2a, b). CSF analysis revealed WBC of 6*10^9^/L, with 100% mononuclear cells, protein and glucose levels were within normal range. The CSF anti-NMDAR antibodies turned negative. CSF oligoclonal IgG bands were negative and the IgG index (4.39 mg/24 h, normal range < 7.0 mg/24 h) fell within normal range. To address the treatment, the patient accepted a second 5-day course of intravenous Immunoglobulin therapy. Continued immune-suppression with mycophenolate mofetil was recommended because anti-NMDAR encephalitis patients without teratoma have a relapse rate as high as 20–25% [11], but was refused by the patient.

She was discharged with close follow-up. Six months later, brain MRI showed markedly reduced abnormality in left basal ganglia area and frontal lobe. The ovarian ultrasound scans remained normal and were scheduled to be taken every 3–6 months.

## Discussion and conclusions

Anti-NMDAR encephalitis is the most common antibody-associated encephalitis and appears to be the leading cause of encephalitis in patients younger than 40 years old [3, 12]. As reported, approximately 45% of anti-NMDAR encephalitis patients over 18 years old and 9% of girls below 14 years old present with ovarian teratomas [11]. Clinical improvement following teratoma removal, anti-NMDAR encephalitis was herein considered paraneoplastic. Recently, several studies demonstrated that viral infection [6] is also a potential cause of anti-NMDAR encephalitis.

Anti-NMDAR encephalitis usually has an acute or subacute onset. Prodromal symptoms such as headache or flu-like symptoms occur frequently and approximately 50% of the anti-NMDAR encephalitis patients present fever during the course of the disease [3, 4, 7]. Neurological manifestations of anti-NMDAR encephalitis were typical of psychosis, behavioral changes, amnesia, decrease levels of consciousness, seizure and movement disorders [4, 7]. Our patient mainly presented with headache, fever, and mild intermediate memory impairment. Cerebrospinal analysis revealed moderate lymphocytic pleocytosis, normal glucose and protein levels. These expressions are highly suggestive of viral or autoimmune encephalitis.

Despite the severity of signs and symptoms, brain MRI alterations are often subtle in more than half of patients [4], and in the remaining patients, abnormalities are present in the white and grey matter. White matter lesions have been reported in the medial temporal, frontal, parietal, occipital lobe, cingulate gyrus and corpus callosum, whereas grey matter lesions have been shown in the cerebral cortex, thalamus and basal ganglia [3, 4, 8–10]. Basal ganglia involvement was detected in less than 5% patients. Adding to the variability, affections of the cerebellum, brainstem and spinal cord have also been observed [4, 13]. Although minimal clinical neurological manifestations were found in our patient, brain MRI revealed diffuse, unenhanced lesions in white matter of the bilateral frontal lobes, left splenium of corpus callosum, and left basal ganglia. The extensive lesions in frontal and temporal lobes, and basal ganglia area, with mild mass effects seen in our patient have not been described previously. In differential diagnoses, demyelinating disorders (such as acute disseminated encephalomyelitis, tumefactive multiple sclerosis), cerebral gliomatosis or metastatic carcinoma were included.

To screen potential neoplasia, F18-FDG-PET was performed. F18-FDG-PET showed hypermetabolism in the cortex of the left frontal, temporal and parietal lobes and that of the right cerebellum, as well as hypometabolism in the left frontal basal ganglia and in the white matter lesions in bilateral frontal lobes. PET-CT results did not indicate any underlying tumors. Previous studies demonstrated hypermetabolism in anterior regions of frontal and temporal lobes compared to relative hypometabolism in posterior regions and hypermetabolism in basal ganglia in anti-NMDAR encephalitis [14, 15]. Our patient presented the same pattern of a relative anterior hypermetabolism in cortex but hypometabolism in left head of caudate nucleus. One possible explanation is that local edema had interrupted metabolism. In our patient, anti-NMDAR encephalitis was confirmed with its positive antibody in CSF. CSF antibody has a higher sensitivity (98.5–100%) in comparison with serum antibody (80.7–89.4%) [16].

Extreme delta brush, the distinctive EEG pattern in anti-NMDAR encephalitis, is present in only 30% of patients, whereas nonspecific slowing and disorganized background activities with or without epileptic waves were seen in most patients [17].

Noting that both clinical and EEG and brain imaging manifestations are nonspecific and that NMDA receptor encephalitis has a rather high occurrence, anti-NMDAR antibody in serum and cerebrospinal fluid should be detected in the patients suspected of cerebral nervous infections, glioma, demyelinating disorders, Hashimoto’s disease, primary or secondary central nervous system vasculitis etc. Once anti-NMDAR encephalitis is confirmed, immunotherapy and teratoma detection and removal should be quickly initiated. First line immunotherapies consist of a combination of corticosteroids and intravenous immunoglobulins, or plasma exchanges. For anti-NMDAR encephalitis patients without remarkable improvement or without tumor, second line immunotherapies, including mycophenolate mofetil, cyclophosphamide and rituximab, should be required [11]. Since the median length of time between symptoms onset and first relapse of anti-NMDAR encephalitis was 2 years, a minimum of 2 years of long-term clinical monitoring is necessary [12].

## Supplementary information

**Additional file 1:** Positive immunofluorescence stain of transfected HEK 293 cells expressing NMDAR NR1α subunites after incubation with the patient's CSF. (JPG 522 kb)

**Additional file 2:** NMDAR immunofluorescence stain of negative control. (JPG 682 kb)

## Data Availability

All data and material supporting our findings are contained within the manuscript.

## Abbreviations

AMPAR: Alpha-amino-3-hydroxy-5-methyl-4-isoxazolepropionic acid receptor
ANA: Antinuclear antibodies
ANCA: Anti-neutrophil cytoplasmic antibodies
AQP-4: Antibody against aquaporin-4
CASPR2: Contactin-associated protein-like 2
CSF: Cerebrospinal fluid
dsDNA: Anti-double-stranded DNA antibodies
EEG: Electroencephalography
FDG: ^18^F-fluorodeoxyglucose
GABA_B_R: Gamma-aminobutyric acid beta receptor
LGI1: Leucine-rich glioma inactivated protein 1
MOG: Myelin oligodendrocyte glycoprotein
MRI: Brain magnetic resonance image
NMDAR: Anti-N-methyl-D-aspartate receptor
PET: Positron emission tomography
