# Supplementary figures and images for: The diffuse involvement of anti-N-methyl-D-aspartate receptor encephalitis in brain: a case report
Source: BMC Neurol. 2019 Sep 26;19:230. doi: 10.1186/s12883-019-1456-6 (PMC6764129; doi:10.1186/s12883-019-1456-6)

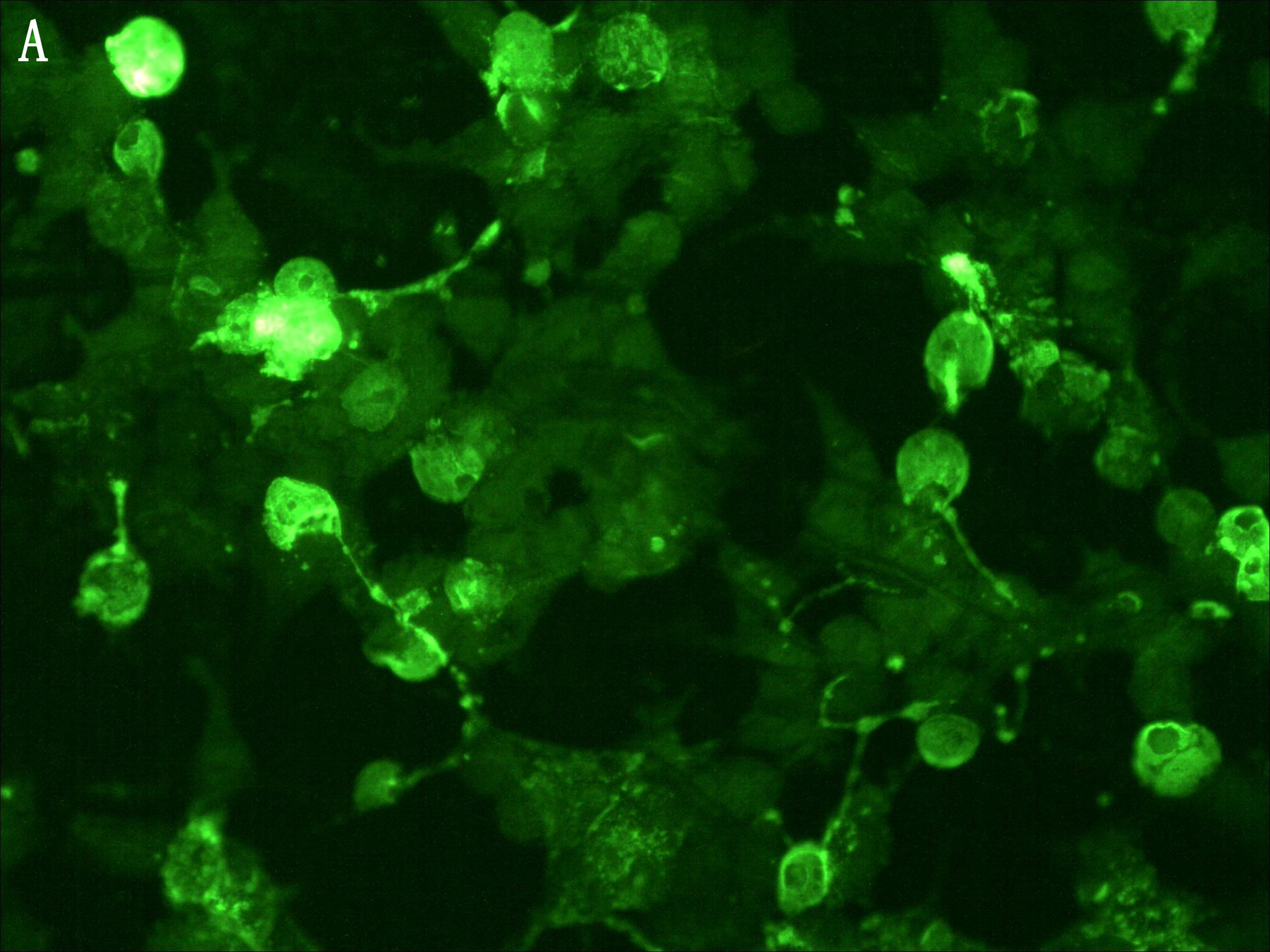

Supplement: Supplementary file 1 — Additional file 1: Positive immunofluorescence stain of transfected HEK 293 cells expressing NMDAR NR1α subunites after incubation with the patient's CSF. (JPG 522 kb) [file 12883_2019_1456_MOESM1_ESM.jpg]

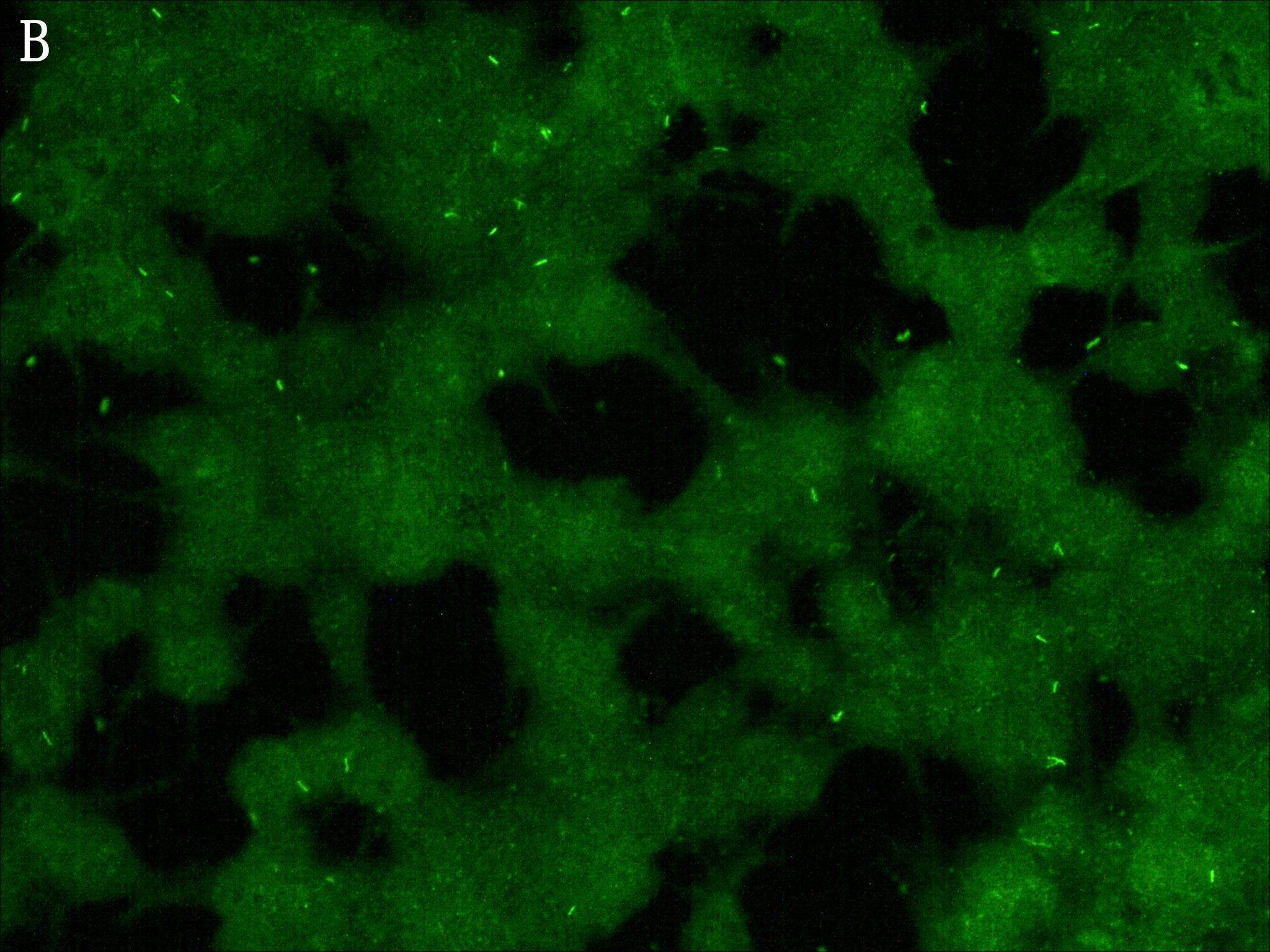

Supplement: Supplementary file 2 — Additional file 2: NMDAR immunofluorescence stain of negative control. (JPG 682 kb) [file 12883_2019_1456_MOESM2_ESM.jpg]
